# Hypoxia lowers *SLC30A8*/ZnT8 expression and free cytosolic Zn^2+^ in pancreatic beta cells

**DOI:** 10.1007/s00125-014-3266-0

**Published:** 2014-05-28

**Authors:** Philipp A. Gerber, Elisa A. Bellomo, David J. Hodson, Gargi Meur, Antonia Solomou, Ryan K. Mitchell, Michael Hollinshead, Fabrice Chimienti, Domenico Bosco, Stephen J. Hughes, Paul R. V. Johnson, Guy A. Rutter

**Affiliations:** 1 Section of Cell Biology, Division of Diabetes, Endocrinology and Metabolism, Department of Medicine, Imperial College London, London, W12 ONN UK; 2 Division of Endocrinology, Diabetes and Clinical Nutrition, University Hospital Zurich, Zurich, Switzerland; 3 Section of Microscopy, Department of Medicine, Imperial College London, London, UK; 4 Mellitech, Grenoble, France; 5 Cell Isolation and Transplantation Centre, Department of Surgery, Geneva University Hospital, Geneva, Switzerland; 6 Nuffield Department of Surgical Sciences, University of Oxford, Oxford, UK; 7 DRWF Human Islet Isolation Facility, Oxford Centre for Diabetes, Endocrinology and Metabolism, Oxford, UK; 8 Oxford NIHR Biomedical Research Centre, Oxford, UK

**Keywords:** Hypoxia, Metallothionein, Type 2 diabetes, Zinc, Zinc transporter

## Abstract

**Aims/hypothesis:**

Hypoxic damage complicates islet isolation for transplantation and may contribute to beta cell failure in type 2 diabetes. Polymorphisms in the *SLC30A8* gene, encoding the secretory granule zinc transporter 8 (ZnT8), influence type 2 diabetes risk, conceivably by modulating cytosolic Zn^2+^ levels. We have therefore explored the role of ZnT8 and cytosolic Zn^2+^ in the response to hypoxia of pancreatic islet cells.

**Methods:**

Human, mouse or rat islets were isolated and exposed to varying O_2_ tensions. Cytosolic free zinc was measured using the adenovirally expressed recombinant targeted zinc probe eCALWY4. Gene expression was measured using quantitative (q)RT-PCR, western (immuno-) blotting or immunocytochemistry. Beta cells were identified by insulin immunoreactivity.

**Results:**

Deprivation of O_2_ (1% vs 5% or 21%) for 24 h lowered free cytosolic Zn^2+^ concentrations by ~40% (*p* < 0.05) and ~30% (*p* < 0.05) in mouse and human islet cells, respectively. Hypoxia similarly decreased *SLC30A8* mRNA expression in islets, and immunoreactivity in beta cells. Implicating lowered ZnT8 levels in the hypoxia-induced fall in cytosolic Zn^2+^, genetic ablation of *Slc30a8* from mouse islets lowered cytosolic Zn^2+^ by ~40% (*p* < 0.05) and decreased the induction of metallothionein (*Mt1*, *Mt2*) genes. Cell survival in the face of hypoxia was enhanced in small islets of older (>12 weeks) *Slc30a8* null mice vs controls, but not younger animals.

**Conclusions/interpretation:**

The response of pancreatic beta cells to hypoxia is characterised by decreased *SLC30A8* expression and lowered cytosolic Zn^2+^ concentrations. The dependence on ZnT8 of hypoxia-induced changes in cell survival may contribute to the actions of *SLC30A8* variants on diabetes risk in humans.

**Electronic supplementary material:**

The online version of this article (doi:10.1007/s00125-014-3266-0) contains peer-reviewed but unedited supplementary material, which is available to authorised users.

## Introduction

Pancreatic beta cells are highly dependent on oxidative metabolism for ATP synthesis, particularly at elevated glucose concentrations [1, 2]. Correspondingly, hypoxia has been shown to influence islet survival and function during transplantation [3, 4]. Moreover, as many as 25% of islets are exposed in vivo to low oxygenation [5], suggesting that hypoxia acts as a regulator of islet function under physiological conditions. Indeed, glucose-induced oxygen consumption creates intracellular hypoxia sufficient to activate hypoxia-inducible factors (HIFs) in rat beta cells [6], an effect that is increased in diabetic animals [7] and which may contribute to defective insulin secretion in some forms of type 2 diabetes.

Hypoxic stress induces genes such as metallothionein (*MT1*/*2*) as a defence against large changes in free metal ion concentration [8], which may affect the activity of anti-oxidative enzymes [9]. Whether genetic factors influence the susceptibility of pancreatic beta cells to hypoxia has not previously been explored. Suggesting this as a possibility, genome-wide association studies (GWAS) have revealed that a non-synonymous single nucleotide polymorphism (rs13266634) in the *SLC30A8* gene (encoding the secretory granule-resident zinc transporter 8 [ZnT8]) is associated with a ~20% increase in disease risk per allele [10–12]. *SLC30A8* expression is largely confined to pancreatic beta and alpha cells [13], and is required for the accumulation of zinc into the secretory granule, where it binds to insulin [14, 15]. Consequently, mice inactivated systemically for *Slc30a8* display profound changes in insulin crystal formation and secretory granule morphology [14, 15], consistent with the lower zinc-transporting activity of the transporter isoform encoded by the risk allele [15]. Defective insulin secretion is seen in global *ZnT8* ^−/−^ mice on some [15], but not all, backgrounds [14, 16, 17], and mice with selectively deleted beta cell *Slc30a8* display marked changes in insulin secretion and glucose tolerance [18]. Whether *Slc30a8* influences cytosolic, as well as granular, Zn^2+^ concentrations has not previously been examined because of the uncertain subcellular targeting of the probes used in earlier work [15].

Monitoring cytosolic Zn^2+^ with the molecularly targeted recombinant probe eCALWY4 [19], the present study aimed to explore the impact of hypoxia on Zn^2+^ homeostasis, and the expression of *SLC30A8*/ZnT8 and other zinc transporters and importers, in human and rodent beta cells.

## Methods

### Reagents

RPMI and CMRL medium, ZnCl_2_, *N*,*N*,*N*′,*N*′-tetrakis(2-pyridylmethyl) ethylenediamine (TPEN), 2-mercaptopyridine *N*-oxide (pyrithione), poly-l-lysine and dimethyloxalylglycine (DMOG) were from Sigma (Gillingham, UK), TRIzol reagent was from Applied Biosystems / Life Technologies (Paisley, UK).

### Mouse and rat strains and maintenance

Female CD1 mice and male Wistar rats were purchased from Harlan (Bicester, UK/Itingen, Switzerland). Global *ZnT8* ^−/−^ mice [15] on a mixed SV129/C57BL/6 background and 129S7/SvEvBrd-*Mt1* ^tm1Bri^ *Mt2* ^tm1Bri^/J mice (purchased from Jackson Laboratory, Bar Harbor, ME, USA) have previously been described [20]. Mice were killed at 10–12 weeks (−15 weeks for cell death analysis) of age by cervical dislocation as approved by the UK Home Office Animal Scientific Procedures Act, 1986 (PPL 70/7349), with ethics approvals.

### Islet isolation, culture, infection and dissociation

Human islets were isolated from six beating-heart donors (electronic supplementary material [ESM] Table 1) with appropriate local ethical permissions (Charing Cross Research Ethics Committee reference 07/H0711/114) in Oxford, UK [21] or in Geneva, Switzerland [22], and maintained in RPMI medium containing 5.5 mmol/l glucose and 10% (vol./vol.) FCS. Mouse and rat islets were prepared as in Ravier and Rutter [23]. Islets were dissociated by 10 min incubation in Hanks’-based enzyme-free cell dissociation buffer (Gibco, Invitrogen, Paisley, UK), plated onto 24 mm sterile coverslips treated with 0.1% poly-l-lysine and allowed to recover overnight in RPMI medium containing 11 mmol/l glucose.

### Hypoxia exposure

Pancreatic islets or beta cells were exposed to hypoxia (1% O_2_, 5% CO_2_, 94% N_2_), normoxia (21% O_2_, 5% CO_2_, 74% N_2_) or other oxygen concentrations using a tissue culture incubator with adjustable O_2_ or a modular incubator chamber (Billups-Rothenberg, del Mar, CA, USA).

### Imaging of free cytosolic Zn^2+^ concentrations

**Fig. 1:**
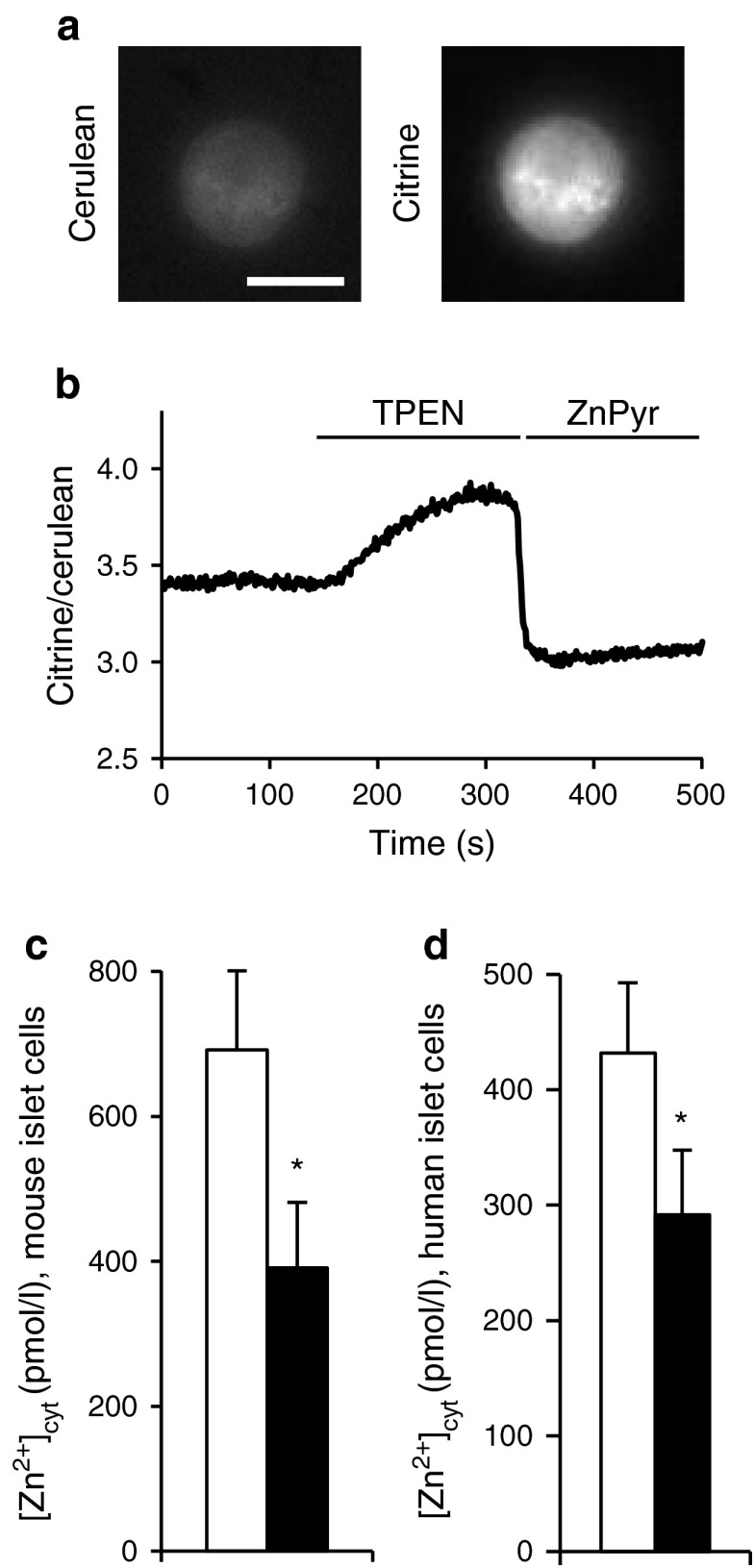
Imaging the effect of hypoxia on [Zn^2+^]_cyt_ in dissociated islets. (**a**) [Zn^2+^]_cyt_ was measured in dissociated CD1 mouse islets as described in the Methods section. Scale bar, 10 μm. (**b**) A representative trace showing changes in citrine/cerulean fluorescence ratio; ZnPyr, 5 μmol/l pyrithione/100 μmol/l ZnCl_2_. (**c**, **d**) Analysis of [Zn^2+^]_cyt_ in dissociated (**c**) CD1 mouse and (**d**) human islets exposed for 24 h to normoxia (white bars) or hypoxia (black bars). Bars represent mean ± SE. ^*^ *p* < 0.05

Adenovirus expressing the recombinant Zn^2+^ probe eCALWY-4 was generated as previously described [24]. Virus was added to dissociated islet cells for 4 h. The medium was then changed, and cells were allowed to express the protein for 48 h. For imaging, cells were washed twice in Krebs Hepes-bicarbonate (KHB) buffer (140 mmol/l NaCl, 3.6 mmol/l KCl, 0.5 mmol/l NaH_2_PO_4_, 0.2 mmol/l MgSO_4_, 1.5 mmol/l CaCl_2_, 10 mmol/l Hepes [pH 7.4], 2 mmol/l NaHCO_3_), 11 mmol/l glucose, pre-equilibrated with 74:21:5 N_2_:O_2_:CO_2_ (normoxia) or 94:1:5 N_2_:O_2_:CO_2_ (hypoxia). Zn^2+^ imaging was carried out as described previously [19], with perifusion of the infected beta cell with buffer subsequently supplemented with 50 μmol/l TPEN and then 5 μmol/l pyrithione/100 μmol/l ZnCl_2_ (ZnPyr) (Fig. 1a, b). For hypoxia-treated cells, care was taken to keep oxygen tension low during microscopy by perifusing cells with oxygen-depleted solutions.

### RNA extraction and qRT-PCR

Total RNA from ~50 islets was obtained using TRIzol reagent and reverse-transcribed into cDNA using a high-capacity cDNA reverse transcription kit (Applied Biosystems). cDNA (equivalent to 10–20 ng of RNA) was subject to qRT-PCR using Power SYBR Green master mix (Applied Biosystems/Life Technologies) in a 7500 fast real-time PCR system (Applied Biosystems/Life Technologies) and analysed by the comparative C_t_ method (primers are shown in ESM Table 2). The expression of target genes was normalised to the expression of cyclophilin A.

### Protein extraction and western (immuno-) blotting analysis

After exposure to normoxia or hypoxia for 24 h, ~200 islets were washed and lysed in ice-cold radioimmune precipitation assay buffer. Total protein extracts (30 μg) were resolved by SDS-PAGE (12% vol./vol. acrylamide) and transferred to PVDF membranes, followed by immunoblotting with rabbit anti-rodent ZnT8 antibody (1:200, Mellitech, Grenoble, France), and mouse monoclonal anti-tubulin (1:5,000, Sigma clone B-5-1-2) antibodies. Secondary horseradish peroxidase (HRP)-linked anti-rabbit antibodies (1:10,000, GE Healthcare, Little Chalfont, UK) were revealed by using ECL detection reagent (GE Healthcare).

### Immunofluorescence

Cells were fixed in 3.7% paraformaldehyde and permeabilised in 0.1% Triton X-100 before immunostaining with a polyclonal anti-swine insulin antibody (1:200, DakoCytomation, Ely, UK), anti-glucagon antibody (G2654, Sigma) and anti-rodent human/rodent ZnT8 antibody overnight at 4°C. Alexa-coupled secondary antibodies (Invitrogen) were used to reveal the primary antibody staining. Confocal imaging was performed as described elsewhere [15].

### Overexpression experiments

INS-1 (832/13) rat beta cells were plated onto 24 mm cover slips and transfected using Lipofectamine 2000 (Invitrogen) with 0.5 μg/well of plasmid encoding eCALWY-4 and 1 μg/well of a construct containing ZnT8 fused in-frame with mCherry (A. Pramatarova, McGill University, Montreal, Canada). At 48 h after transfection, cytosolic Zn^2+^ was imaged as above.

### Dead:live cell assay

Islets were incubated for 15 min in PBS containing 3 μmol/l calcein-AM (Life Technologies) and 2.5 μmol/l propidium iodide (PI; Sigma-Aldrich) before detection of absorbance/emission at 491/525 nm and 561/620 nm, respectively. The islet area occupied by dead cells (PI) was calculated and expressed as a unitary ratio vs that occupied by all (live and dead) cells (PI plus calcein) using ImageJ (http://imagej.nih.gov/ij/) [25].

### Transmission electron microscopy

Isolated islets were fixed and analysed as previously described [26].

### Insulin secretion and content

Total and secreted insulin were measured after batch incubation of islets in KHB buffer as previously described [23].

### Statistics

Data are given as mean ± SE or relative frequency. For comparison of continuous variables in two independent groups, Student’s two-tailed *t* test was used. Bonferroni correction was applied for multiple comparisons. Multiple linear regression was used to assess the effect of different factors on islet cell death. A value of *p* < 0.05 was considered significant. Statistical analyses were performed using SPSS 18.0 software (SPSS, Chicago, IL, USA) and Excel 2010 software (Microsoft, Redmond, WA, USA).

## Results

### Exposure of pancreatic beta cells to hypoxia decreases cytosolic free Zn^2+^ concentrations and Slc30a8/ZnT8 expression

Given the previously described role of Zn^2+^ ions in the responses of other tissues to hypoxia and ischaemia [27], we first determined whether lowered oxygen tensions might affect cytosolic free Zn^2+^ concentrations ([Zn^2+^]_cyt_) in mouse or human islet cells by using eCALWY4. This recombinantly expressed probe is confined exclusively to the cytosolic compartment, and is expected chiefly to report changes in beta cells which predominate in the islet preparations used (>70% for rodent islets [28], ~60% in human islets [29]). A significant lowering in steady-state [Zn^2+^]_cyt_ of 30–40% in preparations from either species maintained under hypoxic conditions (Fig. 1c, d) was detected.

**Fig. 2:**
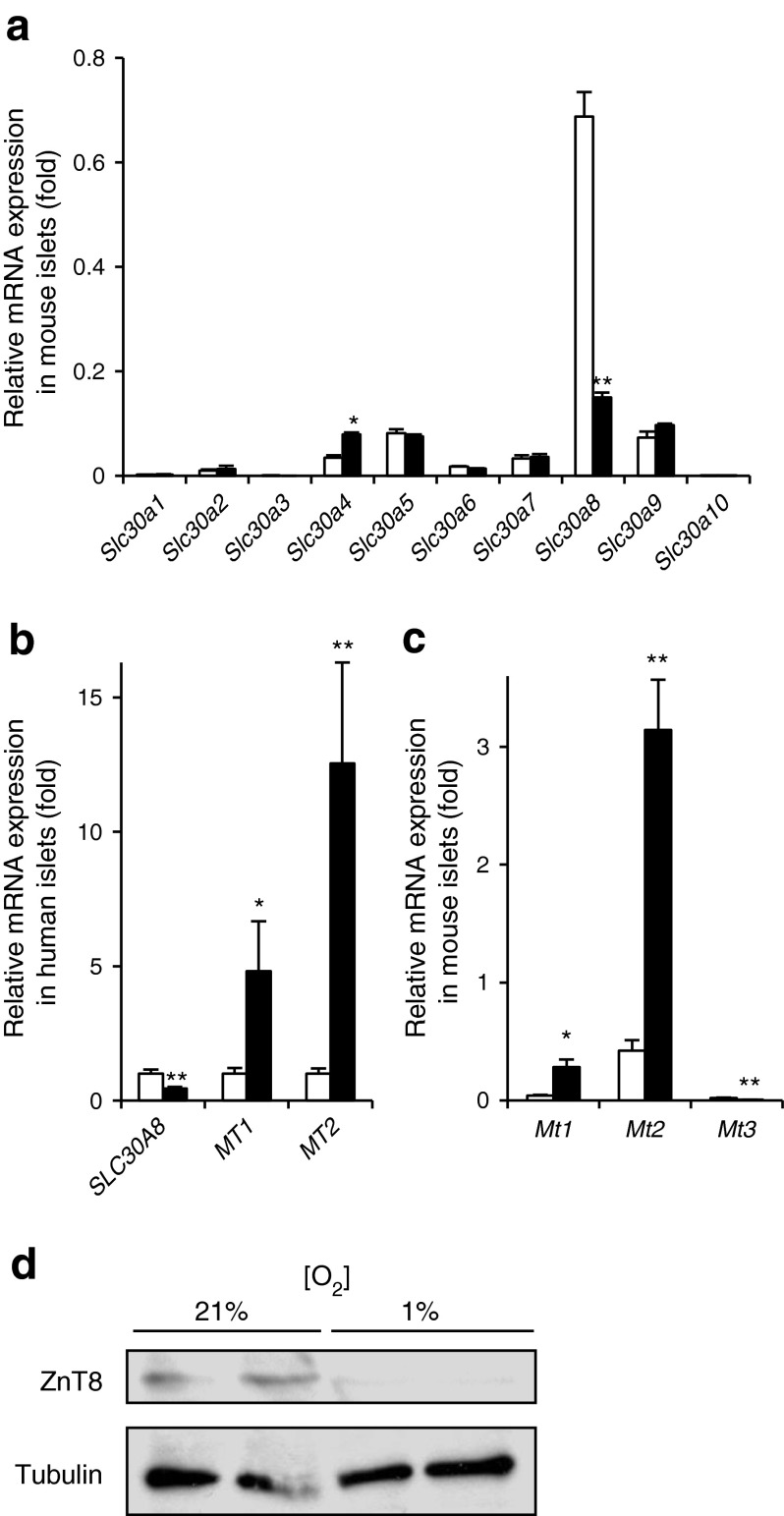
The effect of hypoxia on the expression of genes implicated in Zn^2+^ homeostasis in pancreatic islets. (**a**, **c**) CD1 mouse and (**b**) human islets were incubated for 24 h at normoxia (white bars) or hypoxia (black bars). Expression of (**a**) *Slc30a1*–*10*, (**b**) *SLC30A8*, *MT1*, *MT2* and (**c**) *Mt1*–*Mt3* was determined (normalisation to the expression in normoxia [**b**]). Bars represent mean ± SE. ^*^ *p* < 0.05 and ^**^ *p* < 0.01. (**d**) Western blot analysis of CD1 mouse islets after incubation for 24 h at normoxia (21%) or hypoxia (1%)

**Fig. 3:**
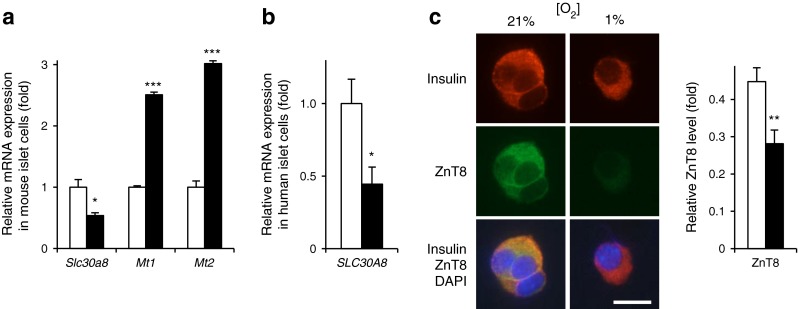
The effect of hypoxia on the expression of the genes implicated in Zn^2+^ homeostasis in dissociated islets. (**a**) Dissociated CD1 mouse and (**b**) human islet cells were incubated for 24 h at normoxia (white bars) or hypoxia (black bars). qRT-PCR analysis of (**a**) *Slc30a8*, *Mt1*, *Mt2* and (**b**) *SLC30A8* was performed (normalisation to the expression in normoxia). (**c**) Dispersed CD1 mouse islet cells were incubated for 24 h at normoxia (21%) or hypoxia (1%). Scale bar, 10 μm. The level of ZnT8 was quantified by calculating the fluorescence ratio of ZnT8 vs insulin (white bar, 21%; black bar, 1%). Bars represent mean ± SE. ^*^ *p* < 0.05, ^**^ *p* < 0.01 and ^***^ *p* < 0.001

To determine whether changes in the expression of key regulators of intracellular Zn^2+^ homeostasis (zinc transporter [*Slc30*/ZnT] and zinc importer [*Slc39*/ZIP] [30] families) are involved in the hypoxia-induced changes in [Zn^2+^]_cyt_, their expression was measured in control or hypoxia-exposed islets. Whereas exposure to hypoxia for 24 h exerted no significant effect on the expression of members of the latter family in both mice and rats (ESM Fig. 1a, b), marked decreases were observed in levels of mRNA encoding *Slc30a8* in both mouse and human islets (Fig. 2a, b), as well as rat islets (ESM Fig. 1c). These changes were accompanied by the expected increase in the expression of the major islet metallothionein genes, *MT1* (also known as *MT1A*) (*Mt1*) and *MT2* (also known as *MT2A*) (*Mt2*) (Fig. 2b, c). Western blot analysis revealed a significant decrease in ZnT8 protein abundance during hypoxia exposure in mouse islets (Fig. 2d). Confirming that the above hypoxia-induced changes were unlikely to require the more severely hypoxic conditions that may exist in the islet core [3], or paracrine intra-islet signalling (e.g. by inflammatory signals) [31], similar changes in the expression of *Slc30a8* and *Mt1*/*2* were apparent in dissociated mouse and human islet cells after exposure to hypoxia for 24 h (Fig. 3a, b). Correspondingly, a hypoxia-induced decrease in the expression of ZnT8 at the protein level was readily revealed by immunocytochemical analysis of individual insulin-positive mouse beta cells (Fig. 3c). To exclude hyperoxia-induced *Slc30a8* overexpression, we compared the effects of 1%, 5% and 21% oxygen concentrations. Exposure to 1%, but not 5%, ambient oxygen altered the expression of *Slc30a8* compared with 21% ambient oxygen (ESM Fig. 2a). The hypoxia-induced changes in *Slc30a8* mRNA levels, as well as cytosolic free Zn^2+^ concentrations in mouse islets/islet cells, were largely reversible after 24 h of re-oxygenation (ESM Fig. 2b, c).

To exclude a decrease in cell viability as the chief reason for the reduced *Slc30a8*/ZnT8 expression, cell death was quantified 24 h after exposure to hypoxia. This analysis revealed a dead:live cell ratio of 12.4 ± 1.9% and 3.9 ± 1.2% in mouse and human islets, respectively, maintained under hypoxic conditions, compared with values of 0.7 ± 0.2 and 0.4 ± 0.1% in normoxia (ESM Fig. 3a, b). Correspondingly, hypoxia did not cause major changes in the morphology or ultrastructure of surviving islet cells as examined by electron microscopy (ESM Fig. 3c). As expected [32, 33], characterisation of hypoxic islets revealed reduced glucose-stimulated insulin secretion: insulin secretion ratio (release at high [16.7 mmol/l] glucose vs low [3.3 mmol/l] glucose) was only 4% of the ratio in normoxia (ESM Fig. 3d). Similarly, KCl-induced insulin secretion was also reduced in hypoxia (ESM Fig. 3d).

**Fig. 4:**
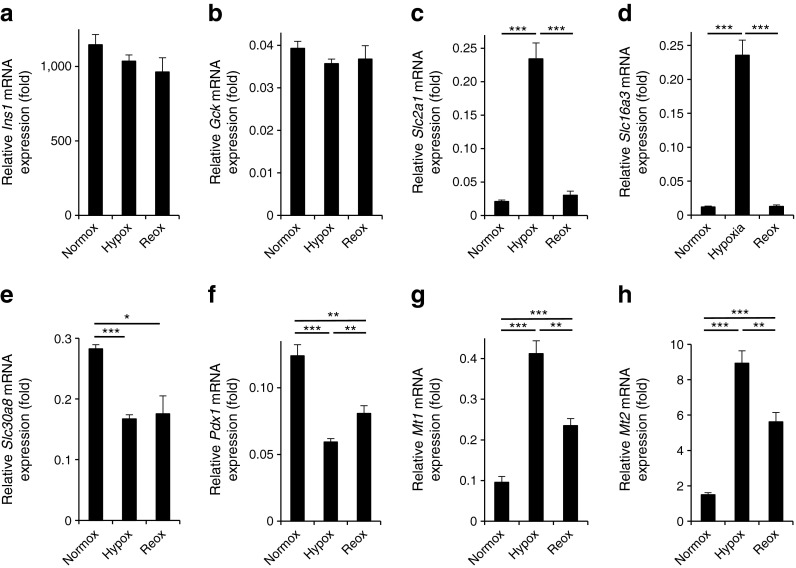
The effect of hypoxia and re-oxygenation on the expression of genes implicated in beta cell differentiation and function as well as Zn^2+^ homeostasis. CD1 mouse islets were incubated at normoxia, at hypoxia for 24 h or hypoxia for 24 h followed by normoxia for 8 h. The qRT-PCR analysis was performed for (**a**) *Ins1*, (**b**) *Gck*, (**c**) *Slc2a1*, (**d**) *Slc16a3*, (**e**) *Slc30a8*, (**f**) *Pdx1*, (**g**) *Mt1* and (**h**) *Mt2*. Hypox, hypoxia; normox, normoxia; and reox, re-oxygenated

We sought next to determine whether the hypoxia-induced changes in the expression of *Slc30a8*/ZnT8 might reflect more global cellular dedifferentiation and/or alterations in the ratio of different islet cell types. Arguing against these possibilities, the expression of beta cell-enriched genes including insulin 1 (*Ins1*) and glucokinase (*Gck*) (also present in alpha cells) [34] did not change significantly during hypoxia and re-oxygenation treatment of mouse islets (Fig. 4a, b). By contrast, and as expected, large changes in the expression of the HIF1α targets GLUT1 (*Slc2a1*) and solute carrier family 16 (monocarboxylic acid transporters), member 3 (MCT4) (*Slc16a3*) were observed (Fig. 4c, d). Similar to the expression of *Slc30a8* (Fig. 4e), expression of pancreatic duodenum homeobox-1 (*Pdx1*), a key transcription factor involved in the regulation of insulin genes and of beta cell differentiation [35], was markedly decreased by hypoxia (Fig. 4f), and this change was not as rapidly reversed as those for the mainly HIF1α-controlled genes (*Slc2a1* and *Slc16a3*) or the metallothioneins (*Mt1*, *Mt2*) (Fig. 4g, h).

To further explore a possible role for HIF1α in controlling *Slc30a8*/ZnT8 expression, pancreatic islets were exposed to the HIF1α-stabilising agent DMOG for 24 h. DMOG treatment did not significantly affect *Slc30a8* expression (ESM Fig. 4).

### Regulation of Slc30a8/ZnT8 by hypoxia does not require changes in metallothionein gene expression

**Fig. 5:**
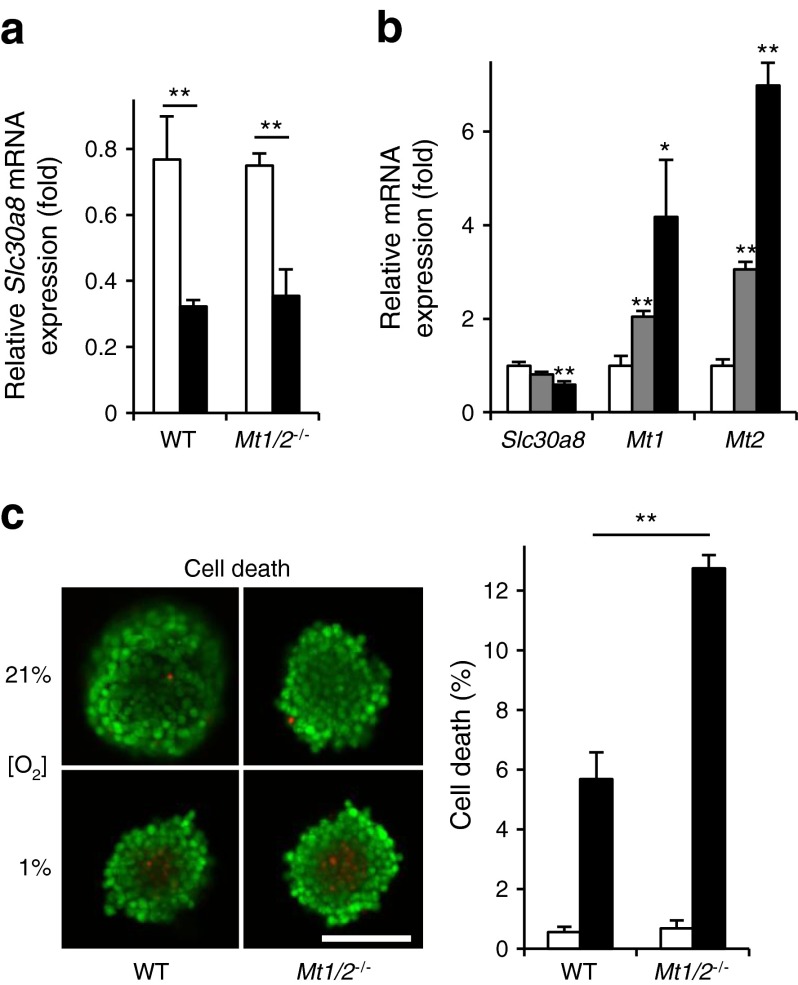
The effect of hypoxia on the expression of *Slc30a8* and cell death in mouse pancreatic islets deficient for metallothioneins 1 and 2. Islets from WT 129S1/SvImJ and 129S7/SvEvBrd-*Mt1* ^tm1Bri^ *Mt2* ^tm1Bri^/J mice were incubated for 24 h at normoxia (white bars) or hypoxia (black bars). (**a**) The qRT-PCR analysis of *Slc30a8* was performed. (**b**) CD1 mouse islets were incubated for 0 h (white bars), 5 h (grey bars) or 10 h (black bars) at hypoxia. qRT-PCR analysis of *Slc30a8*, *Mt1* and *Mt2* was performed (normalisation to the expression in normoxia). (**c**) The percentage of dead cells within intact islets after exposure for 24 h to normoxia (white bars, 21%) or hypoxia (black bars, 1% ambient oxygen). Scale bar, 100 μm. Bars represent mean ± SE. ^*^ *p* < 0.05 and ^**^ *p* < 0.01. *Mt1*/*2* ^−/−^, 129S7/SvEvBrd-*Mt1* ^tm1Bri^ *Mt2* ^*t*m1Bri^/J

The expression of *Slc30a8*/ZnT8 has previously been shown to be regulated by changes in intracellular free Zn^2+^ [36], which in turn are expected to be influenced by alterations in metallothionein expression. We therefore determined whether the regulation of *Slc30a8* expression by hypoxia might be diminished in islets from mice null for *Mt1* and *Mt2* [20]. Hypoxia still resulted in a marked decrease in *Slc30a8* expression in *Mt1* ^*−*/*−*^ *:Mt2* ^*−*/−^ mice, to a similar extent to that observed in wild-type (WT) mouse islets (Fig. 5a). Further supporting a metallothionein-independent effect of hypoxia on *Slc30a8* expression in CD1 mouse islets, *Slc30a8* mRNA levels tended to be decreased as early as 5 h after the initiation of hypoxia (significant decrease after 10 h) (Fig. 5b).

While *Slc30a8* expression was not affected by the loss of *Mt1* and *Mt2*, cell survival after exposure to hypoxia was markedly (~twofold) decreased in *Mt1* ^*−*/*−*^ *:Mt2* ^*−*/−^ islets compared with controls (Fig. 5c).

### Hypoxia-induced metallothionein gene induction and cell death is modulated by ZnT8

**Fig. 6:**
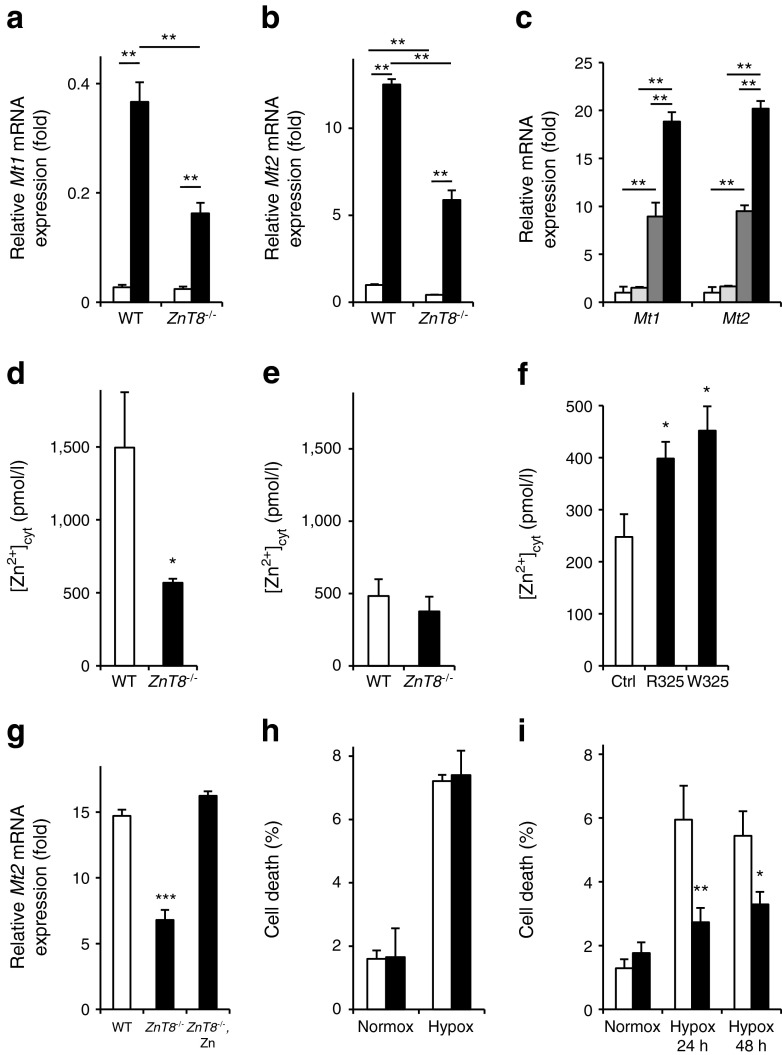
The effect of deficiency for the zinc transporter ZnT8 on the expression of *Mt1* and *Mt2* and [Zn^2+^]_cyt_ as well as cell death rate in mouse pancreatic islets. Islets from *ZnT8* ^+/+^ (WT) and *ZnT8* ^−/−^ mice were incubated for 24 h at normoxia (white bars) or hypoxia (black bars). qRT-PCR analysis of (**a**) *Mt1* and (**b**) *Mt2* was performed. (**c**) CD1 mouse islets were incubated for 24 h at normoxia or hypoxia, with or without 30 μmol/l extracellular ZnCl_2_ (white bars, normoxia without ZnCl_2_; grey bars, normoxia with ZnCl_2_; dark grey bars, hypoxia without ZnCl_2_; black bars, hypoxia with ZnCl_2_); qRT-PCR analysis of *Mt1* and *Mt2* was performed. (**d**, **e**) [Zn^2+^]_cyt_ was measured in dissociated islets of *ZnT8* ^+/+^ (WT) and *ZnT8* ^−/−^ (knockout) mice after incubation for 24 h at (**d**) normoxia or (**e**) hypoxia. (**f**) Analysis of free [Zn^2+^]_cyt_ in INS-1(832/13) cells expressing plasmid constructs encoding *ZnT8* carrying either the at-risk R325 or the protective W325 polymorphism, compared with control (Ctrl). (**g**) Islets from *ZnT8* ^+/+^ (WT, white bars) and *ZnT8* ^−/−^ mice (black bars) were incubated for 24 h at hypoxia, with or without 30 μmol/l extracellular ZnCl_2_ (Zn). qRT-PCR of *Mt2* was performed. (**h**, **i**) The proportion of dead cells in islets of *ZnT8* ^+/+^ (white bars) and *ZnT8* ^−/−^ mice (black bars) is depicted (**h**) after incubation of islets of all sizes for 24 h at normoxia or hypoxia in mice aged 10–15 weeks, and (**i**) for islets <120 μm in older mice (>12 weeks). Bars represent mean ± SE. ^*^ *p* < 0.05, ^**^ *p* < 0.01 and ^***^ *p* < 0.001. Hypox, hypoxia; normox, normoxia; Zn, extracellular ZnCl_2_

To further explore the signalling pathway(s) involved in regulating metallothionein expression and free cytosolic Zn^2+^ in response to hypoxia, we next asked whether inactivation of ZnT8 affected these responses. Induction of *Mt1* and *Mt2* by hypoxia was significantly impaired in *ZnT8* ^−/−^ mouse islets, consistent with the lower [Zn^2+^]_cyt_ in the latter vs WT mouse islet cells (Fig. 6a, b). As expected, *Mt1*/*Mt2* expression was induced by exogenously added Zn^2+^ ions in hypoxia (Fig. 6c). Similar to the observations in hypoxia, [Zn^2+^]_cyt_ was lower in islet cells from mice lacking ZnT8 vs WT islets (Fig. 6d). This difference between WT and *ZnT8* ^−/−^ islets was no longer observed in hypoxia (Fig. 6e). Conversely, overexpression of *Slc30a8* in the clonal INS1 (832/13) beta cell line increased [Zn^2+^]_cyt_ (Fig. 6f). ZnCl_2_ was able to raise the expression of the most abundant metallothionein, *Mt2*, in hypoxic *ZnT8* ^−/−^ mouse islets to levels similar to those of hypoxic WT islets (Fig. 6g). In addition to the regulation of metallothioneins, the expression of other genes important in the response of the beta cell to hypoxia was assessed (ESM Fig. 5). In contrast to metallothionein, there was a tendency for enhanced expression of these genes in *ZnT8* ^−/−^ islets, reaching statistical significance for *Slc16a3* (MCT4) when compared in WT and *ZnT8* ^−/−^ islets during hypoxia. Insulin content did not significantly differ between WT and *ZnT8* ^−/−^ mouse islets in normoxia or hypoxia (ESM Fig. 6).

In contrast to *Mt1* ^*−*/*−*^ *:Mt2* ^*−*/−^ islets, cell survival was not affected in a large sample of *ZnT8* ^−/−^ mouse islets (Fig. 6h) exposed to hypoxia. Multiple linear regression revealed a significant effect of mouse age (*p* = 0.001) and islet size (*p* < 0.001), but not genotype, on islet cell survival. However, when assessed in a defined population of smaller islets (<120 μmol/l) from older mice (12–15 weeks), we observed a significant difference in islet cell survival with a survival advantage of *ZnT8* ^−/−^ mouse islets after 24 h (*p* < 0.001) or 48 h of hypoxia exposure (*p* < 0.01) (Fig. 6i).

## Discussion

We demonstrate here that hypoxia strongly, but reversibly, regulates the expression of *Slc30a8*/ZnT8 in islets and beta cells from human and two rodent species. These findings thus extend the list of pathophysiological factors, currently including cytokines [31, 37] and fatty acids [36], which regulate the expression of this type 2 diabetes risk gene in islets. Hypoxia might therefore contribute to the downregulation of *Slc30a8* previously observed in human type 2 diabetes islets [38].

The present findings extend to the islet beta cell those of a recent report [39] showing that hypoxia lowers the expression of *Slc30a8*/ZnT8 in the retinal pigment epithelium of the eye. The latter studies [39] provided evidence for control of ZnT8 levels via HIF1 stabilisation. By contrast, in our hands, the HIF1α-stabilising agent DMOG tended only slightly to reduce *ZnT8* mRNA levels. Instead, our results suggest that the effects of hypoxia on *Slc30a8*/ZnT8 expression in pancreatic islets are more complex and may conceivably involve changes in the expression of *Pdx1*, recently shown to control *Slc30a8* expression in clonal beta cells [40].

Recently, Lefebvre and colleagues [36] reported that depletion of intracellular zinc reduced *Slc30a8* expression in human islets, confirming findings in INS-1E cells [41]. These earlier observations thus raise the possibility that the lowered cytosolic Zn^2+^ concentrations reported here during hypoxia may contribute to the lowering of *Slc30a8*/ZnT8 expression. Arguing against this view, addition of extracellular Zn^2+^ (30 μmol/l ZnCl_2_) did not rescue the lowering of *Slc30a8* mRNA levels after hypoxia (ESM Fig. 7). Moreover, any direct or indirect (e.g. by alterations in cytosolic Zn^2+^) effect of hypoxia-driven metallothionein gene induction also seems unlikely, as hypoxia-induced changes in *Slc30a8* mRNA levels were not different in *Mt1*/*Mt2* ^−/−^ mice compared with control animals (Fig. 5a).

We propose instead a model by which hypoxia initially depresses *Slc30a8* transcription and/or mRNA stability by mechanisms which remain to be elucidated (as discussed below). This then leads to a lowering of cytosolic free Zn^2+^, and a consequent drop in *Mt1*/*Mt2* expression. The above sequence of events is supported by the observation of reduced cytosolic Zn^2+^ in *ZnT8* ^−/−^ mice compared with controls (Fig. 6d), and by an increased cytosolic Zn^2+^ concentration in clonal pancreatic beta cells when *Slc30a8* was overexpressed (Fig. 6f).

The observation that cytosolic free Zn^2+^ is lowered by ablating *Slc30a8*, a mediator of Zn^2+^ *uptake* into granules, may at first glance appear surprising. We would stress that the use here of a molecularly targeted cytosolic Zn^2+^ probe excludes uncertainties over the subcellular compartment in which Zn^2+^ concentrations are interrogated. So how might this apparent paradox be explained? First, our data suggest that ZnT8 may, under normal circumstances, catalyse Zn^2+^ *efflux* from granules, in line with bidirectional transport by ZnT5 [42]. Alternatively, gradual release of Zn^2+^ from granules by exocytosis may elevate the extracellular Zn^2+^ concentration in the medium surrounding WT, but not *ZnT8* ^−/−^, cells for which granule Zn^2+^ content is near zero (see Chimienti et al and Li et al [13, 43]). The released Zn^2+^ may then be recaptured, at least in part, and taken into the cytosol, by plasma-membrane-located Zn^2+^-uptake systems (e.g. ZIP1, voltage-gated Ca^2+^ channels) [30]. However, we calculate that release of Zn^2+^ ions into the medium is likely to increase total external Zn^2+^ concentration to only a miniscule extent (<50 nmol/l) vs a total extracellular concentration of >6 μmol/l [44], although local concentrations at the cell surface [14, 43] may be higher.

One of the effects of ZnT8 inhibition and consequently lowered cytosolic Zn^2+^ levels was impaired metallothionein induction in response to hypoxia (Fig. 6a, b). However, this did not affect islet cell survival under the conditions examined, despite the fact that islets lacking metallothionein-1 and -2 exhibited a twofold higher frequency of beta cell death compared with control islets (Fig. 5c). The latter finding is consistent with earlier data showing that overexpression of metallothionein protects islets against hypoxia, leading to improved islet cell survival [45]. We assume, therefore, that residual levels of metallothionein (~50%) in *ZnT8* ^−/−^ mouse islets are sufficient to prevent an increase in cell death.

Unexpectedly, in small islets from older mice, we observed a significantly lower rate of cell death in *ZnT8* ^−/−^ compared with WT islets. This finding is in line with our previous report on glucose homeostasis in these mice, where a compromising effect of ZnT8 deficiency disappeared with age [15], and suggests that, with ageing, a deleterious effect of ZnT8 deficiency may revert to one of protection. On the other hand, the lack of a genotype effect in large islets may be due to the variance of oxygen concentration in these islets, where the core is relatively anoxic as a consequence of larger diffusion distance when exposed to hypoxia in vitro. Nonetheless, the expression of hypoxia-inducible genes (with the exception of *Mt1*/*Mt2*) tended to be enhanced in *ZnT8* ^−/−^ mice, possibly reflecting regulation of HIF1α by Zn^2+^ [46]. Importantly, the current results may provide an explanation for the recent finding that rare loss-of-function mutations in the *SLC30A8* gene in man are associated with protection against type 2 diabetes [47].

Reduced expression of ZnT8 in hypoxia may thus reflect an ‘adaptive’ response of beta cells to permit survival under a hypoxic/oxidative stress in a less differentiated state (as previously described after partial pancreatectomy-induced hyperglycaemia [48]). The mechanisms behind this observation remain to be elucidated, but may suggest that reduced zinc levels are beneficial in the situation of increased hypoxic stress. Indeed, it has previously been shown that high concentrations of Zn^2+^ are able to induce islet cell death in a dose-dependent manner [49, 50]. A similar rate of cell death after 24 h and 48 h of hypoxia, as observed here, suggests that, once adapted to hypoxia, islets are better able to survive. Further studies will be required, however, to determine whether pharmacological modification of ZnT8 function may be beneficial in islet transplantation or type 2 diabetes mellitus.

## Electronic supplementary material

Below is the link to the electronic supplementary material.

ESM Table 1 (PDF 3 kb)

ESM Table 2 (PDF 9 kb)

ESM Fig. 1 (PDF 68 kb)

ESM Fig. 2 (PDF 8 kb)

ESM Fig. 3 (PDF 79 kb)

ESM Fig. 4 (PDF 12 kb)

ESM Fig. 5 (PDF 14.3 kb)

ESM Fig. 6 (PDF 4 kb)

ESM Fig. 7 (PDF 11 kb)

## Abbreviations

DMOG: Dimethyloxalylglycine
HIF: Hypoxia-inducible factor
KHB: Krebs Hepes-bicarbonate
MCT4: Solute carrier family 16 (monocarboxylic acid transporters), member 3
PI: Propidium iodide
TPEN: *N*,*N*,*N*′,*N*′-tetrakis(2-pyridylmethyl) ethylenediamine
WT: Wild-type
ZIP: Zinc importer
ZnT8: Zinc transporter 8
