# Supplementary material for: Hypoxia lowers SLC30A8/ZnT8 expression and free cytosolic Zn2+ in pancreatic beta cells
Source: Diabetologia. 2014 May 28;57(8):1635–44. doi: 10.1007/s00125-014-3266-0 (PMC4079946; doi:10.1007/s00125-014-3266-0)
Supplement: Supplementary file 1 — (PDF 3 kb) [file 125_2014_3266_MOESM1_ESM.pdf]

ESM Table 1 - Characteristics of human islet donors

No	Gender	Age (y)	BMI (kg/m^2)
1	F	46	26
2	F	43	34
3	M	20	23
4	M	49	24
5	M	46	36
6	M	52	27
