# Supplementary material for: Hypoxia lowers SLC30A8/ZnT8 expression and free cytosolic Zn2+ in pancreatic beta cells
Source: Diabetologia. 2014 May 28;57(8):1635–44. doi: 10.1007/s00125-014-3266-0 (PMC4079946; doi:10.1007/s00125-014-3266-0)
Supplement: Supplementary file 2 — (PDF 9 kb) [file 125_2014_3266_MOESM2_ESM.pdf]

ESM Table 2 - Primer sequences used for qRT-PCR

**Target**	**Mouse (for/rev)**	**Rat (for/rev)**	**Human (for/rev)**
Slc30a1	ACTCAGTGTGCCCTGAAGCA<br>GCATCCTTCCCAGAATGCA	CCCAGCTTCATACATGCAGGTG<br>CCTTGCTCTTCTCCCCTATATGCT	
Slc30a2	CCCGACCAGCCACCAA<br>CCAAGGATCTCGGCTCGAT		
Slc30a3	GGTGGTTGGTGGGTATTTAGCA<br>CAAGTGGGCGGCATCAGT	TCCTCTTCTCTATCTGCGCCC<br>TGTGCGGAGGCAACGTGGTAA	
Slc30a4	CATCGCTGCCGTCCTCTAC<br>ATTTGCCATGTATCCACCTACAAG	GATCGGAGAGCTTGTAGGTGGATA<br>ACACCAGCATGACACTGATCATGG	
Slc30a5	CTGTTTGCTGCCCTGATGAG<br>CGGCCATACCCATAGGAGAA		
Slc30a6	TGAGCCCGGTTATTCTTCTGA<br>CCGCGATTAAGACCCAAACTAT	GCTGACCGAAGGTCCTGGAAGA<br>TAGGCAGCGCTAGGTCTCCTCAC	
Slc30a7	ATGTTGCCCCTGTCCATCAAGG<br>TCGGAGATCAAGCCTAGGCAGT	ATGTTGCCCCTGTCCATCAAGG<br>TCGGAGATCAAGCCTAGGCAGT	
Slc30a8	CGCAGTTGATGGCGTGATC<br>TGGTTCACAGTCAGTGACCAGAT	GGTGGACATGTTGCTGGGAG<br>CACCAGTCACCACCCAGATG	TGGCCACAATCACAAGGAAGT<br>CAAGGGCATGCACAAAAGC
Slc30a9	GTAGGGACATTGGCTGCTTAGG<br>TTCAACACAGGCTGGCTGAA		
Slc30a10	TTTCCCCTCCCTGGATCAG<br>ACACAGGCTCAAAGAAACGAGAT		
Slc39a1	CAGACGTGGTCAGGGACATTAG<br>TGTACTGCCTGGCCTCTGACT		
Slc39a2	TGGCGGCCACCATACAG<br>AACACCACAAGCCCCTTATG	GTCTCTGCTTACCCTACACC<br>CTTCCTGTACTATTCTGCACCA	
Slc39a3	TGTCAGCTTCTCCTATGGCTTGT<br>GGATCCCGCCTGCACTAATA	GAGGAGAATACATAAGAACAACCC<br>TCTGCTCAGCAGCTTGAG	
Slc39a4	GGGCCGTGTGAAAAGTGTCT<br>GGCTTGTCAGGTTTGCCTAGA	TCTTCAACCTCTTATTGCCCA<br>CTCCTCTGTCACCAAGTCTG	
Slc39a5	CGAGCTGCCCCACGAA<br>AAAGGATAACCCTTCCTGAAGCA	TAGACTCAGGTACAATCCTCAG<br>GCAGTCAATGTCCCATTCTC	
Slc39a6	ACATTGGCCTGGATGGTGAT<br>AAGGCCGTCACTGAAATTGTG	AGTTTAACTATCTCTGTCCAGCC<br>CTTGCATGAGAATGTGGGAG	
Slc39a7	GCTGGGTCCTGCCATTCA	GGATTGTCGCCTTTCTTGTG	

	CAGCACAGACACTGTTGCTACGT	AAGGACACTCATGCCTATCC	
Slc39a8	GTACGCAGGAGACATCGAATTG<br>TGCCTTCCCGCGTTGA	TGGCAGATATGTTTCCAGAG<br>CAAGGTGATGAGCAAGATGG	
Slc39a9	GGACCAGGCTGGCTTCAAA<br>CCAGCTCTAAGGAGGCAGAAAC	AGCAGCAGAAATATCAGTTGTC<br>TGATTCTGGATCGTCAGTGG	
Slc39a10	GCCCTTCACCAGAGACCAATAA<br>CCTCCTGACCTTCACTGACTTCA	CCAACATGAATGTTTGAACGTC<br>AAGGAGAGCAGACTAATAACGG	
Slc39a11	CTCACCTGGGTGCTACAGAAGAC<br>CAATGCAGGGTCCAAGTTCA	ATGCTTCAAGGTTACAGCTC<br>ATCTAAGATCCGCCTCTGC	
Slc39a12	GGTTGTAAATTTGTCCTGCATGAA<br>TTGGGCTTGGGTTGTGTTG	TATTTCTTCAGAACCACAGCAG<br>CGTGCTATAACCATATTTCTCCAG	
Slc39a13	AGGAATGTCAACTGGAAGAATGC<br>GGTGTCAGCCAAGGCAAATAGT	CAAAGGAGTAGAGGAGACGG<br>CATCTTCCTCCAAGAGGTCAG	
Slc39a14	GAGTGGGCCGGGATAATGTT<br>AAAGCACGTGGAGAGGTTCCT		
Mt-1	CTGCACTTGCACCAGCTC<br>GGAGCAGCAGCTCTTCTTG		CTCGAAATGGACCCCAACT<br>ATATCTTCGAGCAGGGCTGT
Mt-2	AAGAAAAGCTGCTGCTCCTG<br>CACTTGTCGGAAGCCTCTTT		CCGACTCTAGCCGCCTCTT<br>GTGGAAGTCGCGTTCTTTACA
Mt-3	CCCTGCCGGATGTGAGAA<br>CCCCCTCTTCACCTTTGCA		
Ins1	GAAGCGTGGCATTGTGGAT<br>TGGGCCTTAGTTGCAGTAGTTCT		
Gck	GCTTTTGAGACCCGTTTTGTG<br>GCCTTCGGTCCCCAGAGT		
Slc2a1	CAGTTCGGCTATAACACTGGTG<br>GCCCCCGACAGAGAAGATG		
Slc2a2	CATGCTGAGCTCTGCTGAAG<br>ACAGTCCAACGGATCCACTC		
Pfk	ATGGCAAAGCTATCGGTGTC<br>ACACAGTCCCATTTGGCTTC		
Pdk1	GGACTTCGGGTCAGTGAATGC<br>TCCTGAGAAGATTGTCGGGGA		
Ldha	TGTCTCCAGCAAAGACTACTGT<br>GACTGTACTTGACAATGTTGGGA		
Slc16a3	GGCTGTTTTATCATCACGGGTT<br>GTGTCGCTGTAGCCAATCCC		

Vegfa	GTACCTCCATGCCAAGT<br>ACTCCAGGGCTTCATCGTTA		
Pdx1	CATCCTATCCTCGGCCAATG<br>CATTCGGTTGCCCAGGAA		
Cyclophillin A	TATCTGCACTGCCAAGACTGTA<br>CCACAATGCTCATGCCTTCTTTCA		
