# Supplementary figures and images for: Hypoxia lowers SLC30A8/ZnT8 expression and free cytosolic Zn2+ in pancreatic beta cells
Source: Diabetologia. 2014 May 28;57(8):1635–44. doi: 10.1007/s00125-014-3266-0 (PMC4079946; doi:10.1007/s00125-014-3266-0)

ESM Figure 1

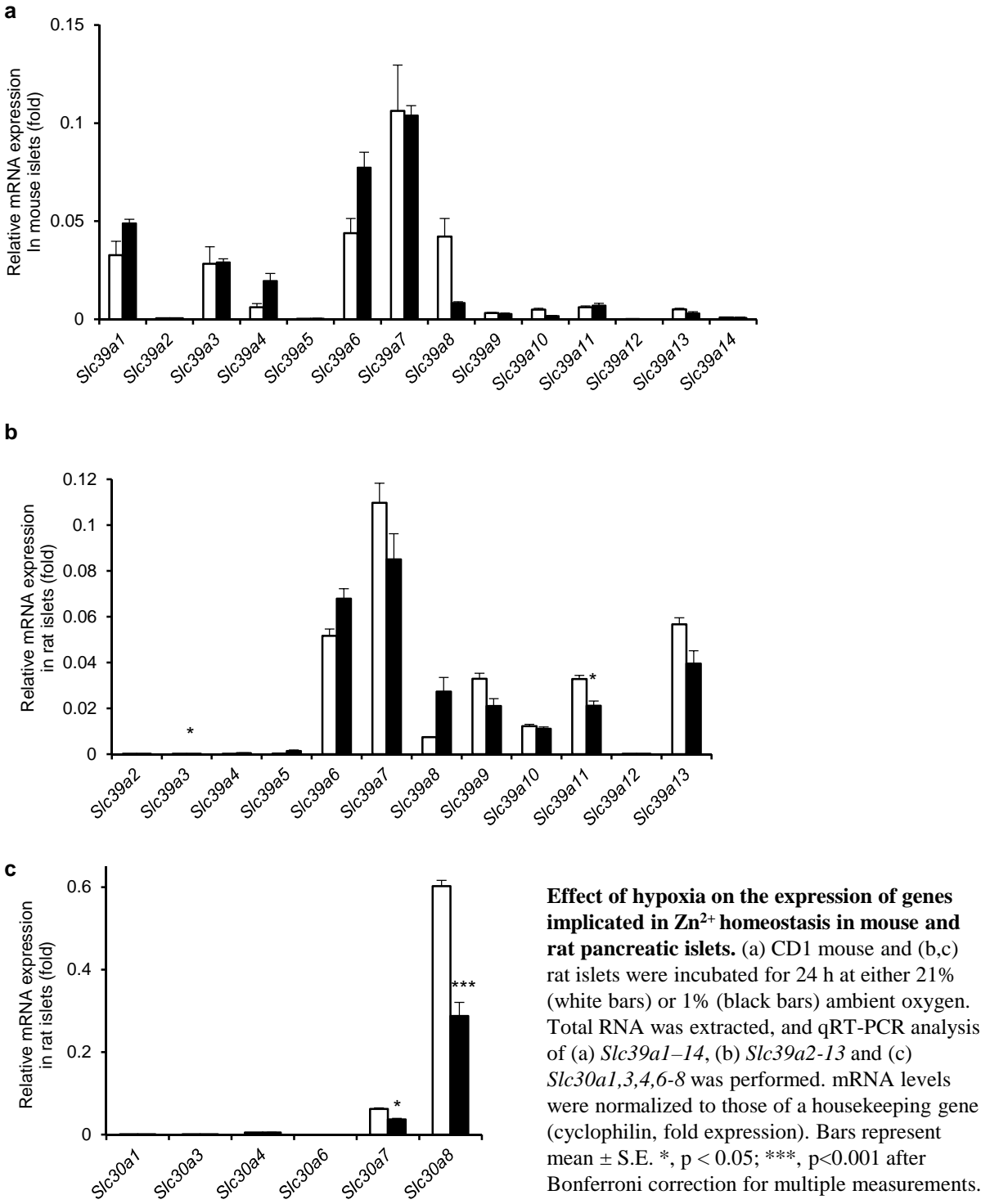

Supplement: Supplementary file 3 — (PDF 68 kb) [file 125_2014_3266_MOESM3_ESM.pdf]
