# Supplementary material for: Hypoxia lowers SLC30A8/ZnT8 expression and free cytosolic Zn2+ in pancreatic beta cells
Source: Diabetologia. 2014 May 28;57(8):1635–44. doi: 10.1007/s00125-014-3266-0 (PMC4079946; doi:10.1007/s00125-014-3266-0)
Supplement: Supplementary file 4 — (PDF 8 kb) [file 125_2014_3266_MOESM4_ESM.pdf]

ESM Figure 2

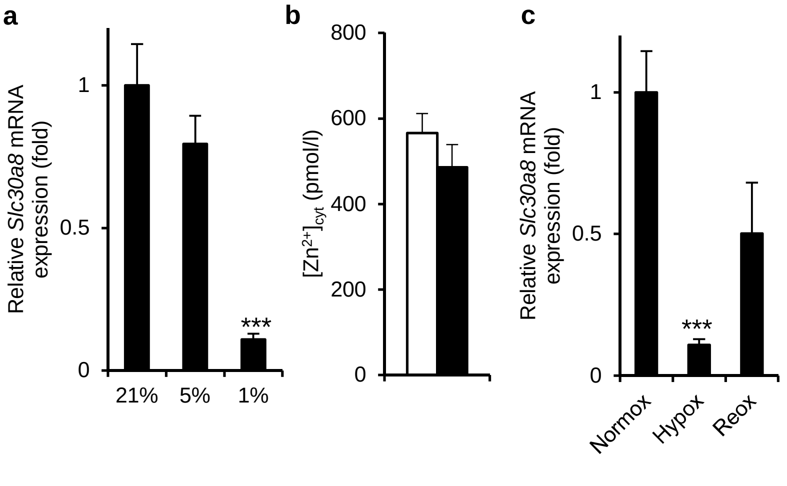

Effects of different Oxygen concentrations and reoxygenation on ZnT8 expression and cytosolic Zn^{2+} concentration. (a) CD1 mouse islets were incubated at either 21%, 5% or 1% ambient oxygen for 24 h. Total RNA was analysed using qRT-PCR. Expression of *Slc30a8* was determined (fold expression, normalisation to cyclophilin). (b) Dissociated CD1 mouse islets were incubated at either 21% ambient oxygen (white bars) or at 1% ambient oxygen for 24 h with consecutive reoxygenation (21%) for 24 h (black bar). $[Zn^{2+}]_{cyt}$ was measured after infection with eCALWY-4-expressing adenovirus. (c) CD1 mouse islets were incubated at either 21% ambient oxygen (Normox), 1% ambient oxygen for 24 h (Hypox) or 1% ambient oxygen for 24 h with consecutive reoxygenation (21% oxygen) for 24 h (Reox). Total RNA was analysed using qRT-PCR. Expression of *Slc30a8* was determined (normalisation to cyclophilin). Bars represent mean ± S.E. ***, $p < 0.001$.
