# Supplementary material for: Hypoxia lowers SLC30A8/ZnT8 expression and free cytosolic Zn2+ in pancreatic beta cells
Source: Diabetologia. 2014 May 28;57(8):1635–44. doi: 10.1007/s00125-014-3266-0 (PMC4079946; doi:10.1007/s00125-014-3266-0)
Supplement: Supplementary file 5 — (PDF 79 kb) [file 125_2014_3266_MOESM5_ESM.pdf]

ESM Figure 3

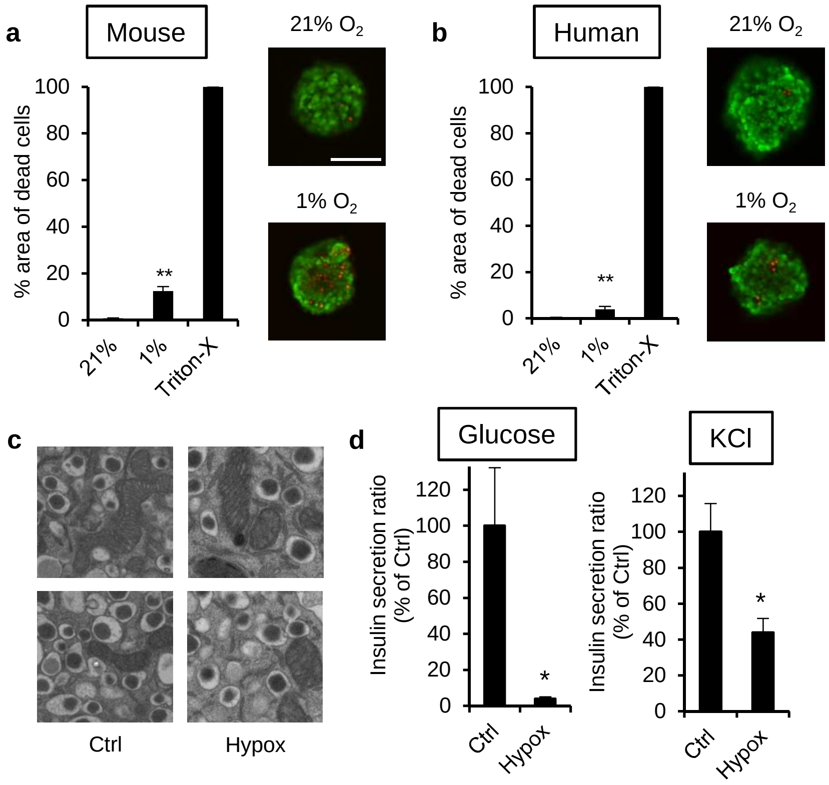

Effect of hypoxia on cell death, cellular ultrastructure and insulin secretion. (a) CD1 mouse and (b) human islets were incubated for 24 h at either 21%, 1% ambient oxygen, or treated with 1% Triton X-100 for 5 min. The percentage of dead cells within intact islets was assessed using calcein and propidium iodide staining. Scale bar, 100 µm. (c) Electron micrographs of CD1 mouse islets after incubation for 24 h at either 21% or 1% ambient oxygen. (d) After 24 h of exposure to 21% (Ctrl) or 1% (Hypox) ambient oxygen, insulin secretion from CD1 mouse islets was assessed during exposure to high glucose (16.7 mM) or KCl (30mM), and the ratio of insulin secretion (fold change *versus* basal secretion at 3 mM glucose) was calculated and expressed as % of Ctrl. Bars represent mean ± S.E. *, $p < 0.05$; **, $p < 0.01$
