# Supplementary material for: Hypoxia lowers SLC30A8/ZnT8 expression and free cytosolic Zn2+ in pancreatic beta cells
Source: Diabetologia. 2014 May 28;57(8):1635–44. doi: 10.1007/s00125-014-3266-0 (PMC4079946; doi:10.1007/s00125-014-3266-0)
Supplement: Supplementary file 6 — (PDF 12 kb) [file 125_2014_3266_MOESM6_ESM.pdf]

**ESM Figure 4**

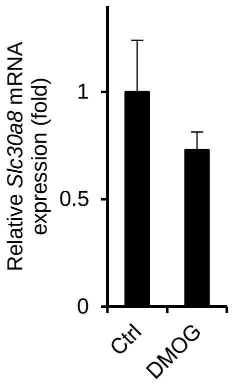


**Effect of HIF-1a stabilization on the expression of *Slc30a8*.** CD1 mouse islets were incubated in the presence of 2mM Dimethyloxalylglycine (DMOG) to induce stabilization of HIF-1α in normoxia. Total RNA was extracted, and qRT-PCR analysis of *Slc30a8* was performed. The mRNA levels were normalized to those of a housekeeping gene (cyclophilin) and to the expression in islets not exposed to DMOG (control, Ctrl). Bars represent mean ± S.E. p (ns).
