# Supplementary material for: Hypoxia lowers SLC30A8/ZnT8 expression and free cytosolic Zn2+ in pancreatic beta cells
Source: Diabetologia. 2014 May 28;57(8):1635–44. doi: 10.1007/s00125-014-3266-0 (PMC4079946; doi:10.1007/s00125-014-3266-0)
Supplement: Supplementary file 7 — (PDF 14.3 kb) [file 125_2014_3266_MOESM7_ESM.pdf]

ESM Figure 5

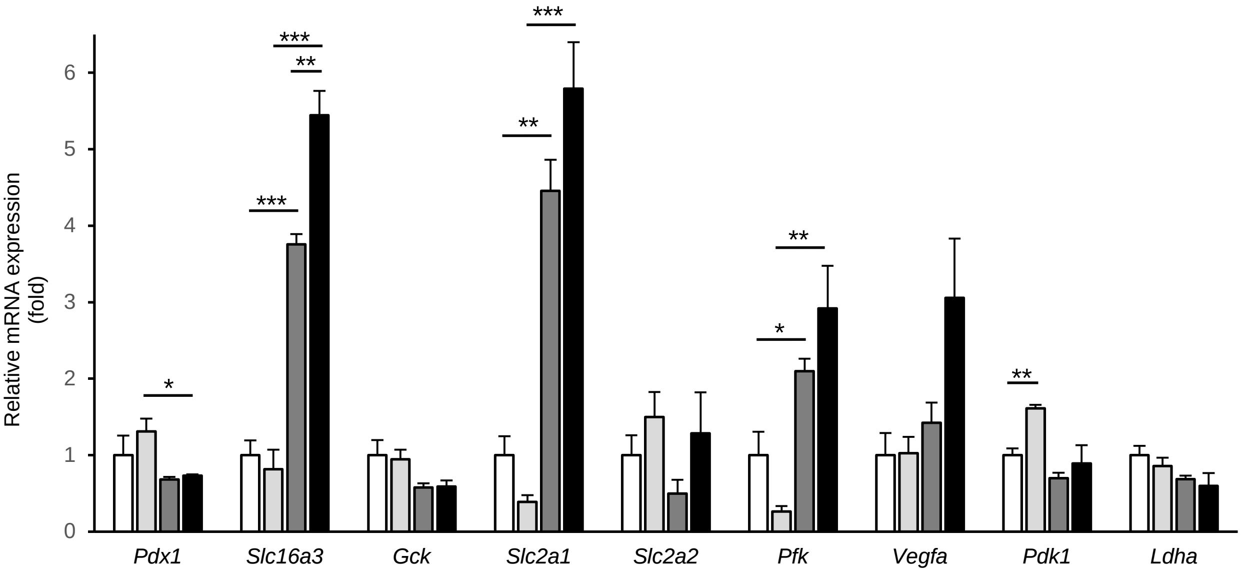

Effect of ZnT8 deletion and hypoxia on the expression of genes involved in glucose mediated insulin secretion and the response to hypoxia in pancreatic beta cells. $ZnT8^{+/+}$ and $ZnT8^{-/-}$ mouse islets were incubated at either 21% ambient oxygen (white bars $ZnT8^{+/+}$, grey bars $ZnT8^{-/-}$) or 1% ambient oxygen (dark grey bars $ZnT8^{+/+}$, black bars $ZnT8^{-/-}$) for 24 hours. Total RNA was extracted, and qRT-PCR analysis of *Pdx1, Slc16a3, Gck, Slc2a1, Slc2a2, Pfk* (Phosphofructokinase), *Vegfa* (Vascular endothelial growth factor A), *Pdk1* (Pyruvate dehydrogenase kinase 1) and *Ldha* (Lactate dehydrogenase A) was performed (fold expression, normalisation to cyclophilin). Bars represent mean ± S.E. *, $p < 0.05$; **, $p < 0.01$; ***, $p < 0.001$
