# Supplementary material for: Hypoxia lowers SLC30A8/ZnT8 expression and free cytosolic Zn2+ in pancreatic beta cells
Source: Diabetologia. 2014 May 28;57(8):1635–44. doi: 10.1007/s00125-014-3266-0 (PMC4079946; doi:10.1007/s00125-014-3266-0)
Supplement: Supplementary file 8 — (PDF 4 kb) [file 125_2014_3266_MOESM8_ESM.pdf]

ESM Figure 6

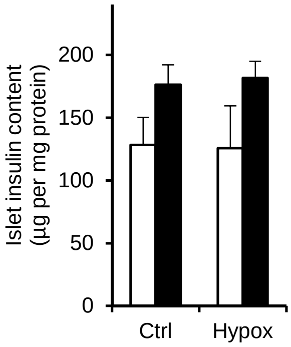

Effect of hypoxia on insulin content. Insulin content in ZnT8$^{+/+}$ (white bars) and ZnT8$^{-/-}$ mice (black bars) was measured by acid alcohol extraction after 24 h of exposure to 21% (Ctrl) or 1% (Hypox) ambient oxygen. Bars represent mean ± S.E., n.s.
