# Supplementary material for: Hypoxia lowers SLC30A8/ZnT8 expression and free cytosolic Zn2+ in pancreatic beta cells
Source: Diabetologia. 2014 May 28;57(8):1635–44. doi: 10.1007/s00125-014-3266-0 (PMC4079946; doi:10.1007/s00125-014-3266-0)
Supplement: Supplementary file 9 — (PDF 11 kb) [file 125_2014_3266_MOESM9_ESM.pdf]

ESM Figure 7

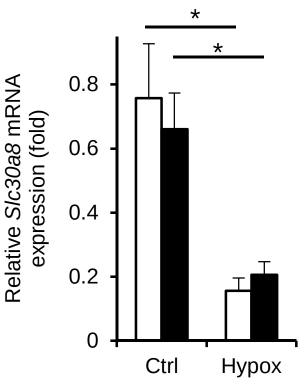

Effect of $ZnCl_2$ on the expression of *Slc30a8*. CD1 mouse islets were incubated in the presence of 0 (white bars) or 30 μM (black bars) $ZnCl_2$ during normoxia (Ctrl) or 1% ambient oxygen (Hypox) for 24 h. Total RNA was extracted, and qRT-PCR analysis of *Slc30a8* was performed. The mRNA levels were normalized to those of a housekeeping gene (cyclophilin). Bars represent mean ± S.E. *, $p < 0.05$
